# Computed Tomography Imaging Characteristics in the Diagnosis and Assessment of Cellulitis in Patients with Leg Swelling

**DOI:** 10.3390/medicina61060982

**Published:** 2025-05-26

**Authors:** In-Chul Nam, Doo-Ri Kim, Jeong-Sub Lee, Jeongjae Kim, Young-Heun Shin, Jung-Ho Won, Yong-June Lee

**Affiliations:** 1 Department of Radiology, Jeju National University School of Medicine, Jeju National University Hospital, 15, Aran 13-gil, Jeju 63241, Republic of Korea; antisors@naver.com (D.-R.K.); shinshlee@naver.com (J.-S.L.); jsquare8057@gmail.com (J.K.); 2 Department of Surgery, Jeju National University School of Medicine, Jeju Natuional University Hospital, 15, Aran 13-gil, Jeju 63241, Republic of Korea; youngheunshin@gmail.com; 3 Department of Radiology, Gyeongsang National University College of Medicine, Gyeongsang National University Hospital, Jinju 52727, Republic of Korea; circlehoya@naver.com; 4 Department of Internal Medicine, Inje University College of Medicine, Haeundae Paik Hospital, Haeundae 48108, Republic of Korea; mouse195@naver.com

**Keywords:** computed tomography, swelling, cellulitis, sensitivity

## Abstract

*Background and Objectives:* This study aimed to evaluate the imaging characteristics of computed tomography (CT) for diagnosing and assessing cellulitis in patients with leg swelling. *Materials and Methods:* Overall, 1545 patients who underwent CT for leg swelling were retrospectively analyzed. Among them, 174 were diagnosed with cellulitis based on clinical findings, laboratory tests indicating inflammation, and their response to antibiotics. Patients with previous follow-up CT scans, negative CT findings, or alternative diagnoses were excluded (*n* = 1123). Two radiologists independently assessed the CT images while blinded to other clinical data, resolving discrepancies through consensus. *Results:* Among 174 patients with cellulitis, 112 were men, and 62 were women, with a mean age of 64.1 ± 15.8 years. The patients were categorized into two groups based on the affected region: Group I (thigh or lower leg, *n* = 130) and Group II (foot involvement only, *n* = 44). The qualitative CT features analyzed included skin thickening (83.9%), subcutaneous edema (94.8%), early venous return (82.8%), lymph node pathological findings (72.4%), and prominent lymph node enhancement (79.3%). Group I demonstrated significantly higher rates of skin thickening, subcutaneous edema, and early venous return than Group II. Pairwise analysis of CT findings revealed significant associations between skin thickening and subcutaneous edema, skin thickening and early venous return on MIP, and early venous return on MIP and lymph node pathology. *Conclusions:* CT provides valuable diagnostic features of cellulitis in patients with leg swelling. Skin thickening, subcutaneous edema, early venous return, and lymph node abnormalities are key findings in the diagnosis of cellulitis.

## 1. Introduction

Cellulitis, an acute non-necrotizing bacterial infection of the skin and underlying soft tissues, remains a common clinical challenge in outpatient and inpatient settings [1,2,3,4]. Characterized by redness, swelling, warmth, and pain, cellulitis can progress rapidly if left untreated, potentially leading to severe complications such as abscess formation, septicemia, and even necrotizing fasciitis [1,2,3,4]. Therefore, prompt diagnosis is crucial to avoid disease progression and complications. Traditionally, the diagnosis of cellulitis has largely been clinical and based on physical examination and patient history. However, the hallmark symptoms of cellulitis, including redness, swelling, warmth, and tenderness, are non-specific and vary in severity. Moreover, its clinical presentation overlaps with a broad spectrum of conditions that cause lower extremity swelling, often complicating an accurate diagnosis [5,6,7,8]. These include deep vein thrombosis (DVT), lymphedema, chronic venous insufficiency, inflammatory arthritis, etc. Misdiagnosis is not uncommon, especially in elderly patients or those with chronic comorbidities, leading to either unnecessary antibiotic use or delayed treatment of alternative diagnoses. In such ambiguous cases or when complications are suspected, imaging can play a pivotal role in rapidly progressing cellulitis or evaluating the extent of infection [9,10]. Various imaging modalities such as ultrasound and magnetic resonance imaging (MRI) have been used to assess skin and soft tissue infections [9,10,11,12]. Among imaging modalities, ultrasound is often used as a first-line tool due to its accessibility and ability to evaluate superficial tissue involvement and vascular conditions. However, it may be limited in obese patients or when deep extension is suspected [13,14,15]. MRI provides excellent soft-tissue contrast and is useful for evaluating deep infections and differentiating cellulitis from abscess or necrotizing fasciitis, but it is often time-consuming and less available in urgent care settings [16,17,18]. Computed tomography (CT), on the other hand, offers several practical advantages, including rapid acquisition, excellent spatial resolution, and the ability to visualize deeper structures and detect early complications. It is particularly useful in emergency settings where quick decision-making is critical [19,20]. However, the precise role and utility of CT in diagnosing and managing cellulitis have been less explored compared to its counterparts. Moreover, the influence of anatomical distribution on CT findings—such as whether the infection involves the thigh, calf, or foot—has not been well studied. Given that tissue composition and lymphatic drainage vary by region, it is plausible that imaging manifestations may differ accordingly.

In this study, we aimed to evaluate the characteristic CT findings associated with cellulitis and to explore potential differences in CT features based on anatomical involvement, particularly between proximal (thigh or lower leg) and distal (foot-only) cellulitis, where CT may offer invaluable diagnostic clarity.

## 2. Materials and Methods

### 2.1. Study Population

The Institutional Review Board of our hospital approved this study, and the requirement for informed consent was waived owing to the retrospective nature of the study. We retrospectively reviewed our institutional picture archiving and communication system. Using the search term “leg swelling”, 1545 patients who underwent lower extremity CT angiography between 1 January 2015 and 30 June 2023 were identified. Among these 1545 patients, we included those with cellulitis, defined as patients whose leg edema improved after antibiotic therapy and who exhibited prominent inflammatory markers on laboratory tests, such as elevated white blood cell (WBC) count, C-reactive protein (CRP) level, and erythrocyte sedimentation rate (ESR). Additionally, patients were required to present with inflammatory symptoms, including redness, pain, warmth, and swelling.

**Figure 1:**
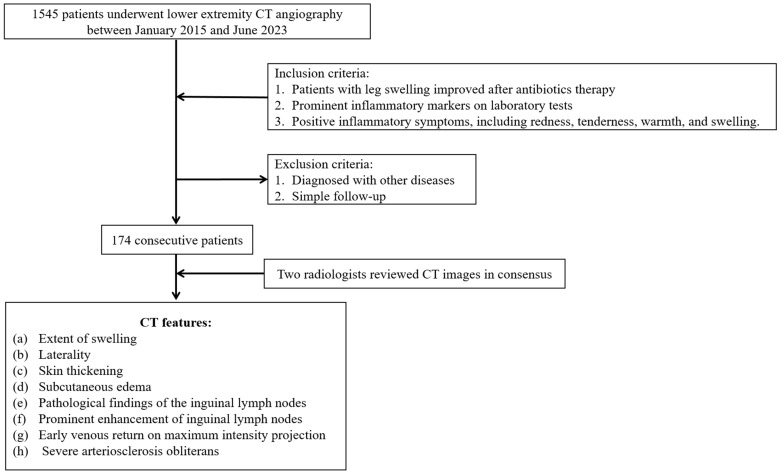
Case accrual process.

We excluded patients with other primary causes of leg swelling, such as generalized edema, lymphedema, deep vein thrombosis, hematoma, idiopathic leg swelling, chronic venous stasis, heart failure, cirrhosis, and renal failure when these conditions were considered the main cause of swelling at the time of CT imaging. Patients with these comorbidities were not excluded if cellulitis was clinically determined to be the primary cause of leg swelling. We also excluded those who had undergone CT examination for previous follow-up purposes. Patients were classified into two groups based on the anatomical involvement of cellulitis. Group I included patients with proximal cellulitis involving the thigh or lower leg with or without foot involvement. Group II included patients with cellulitis limited to the foot without extension to the calf or thigh. Figure 1 illustrates the accrual process.

### 2.2. CT Protocol

Two multi-detector CT scanners were used to perform the lower-extremity CT scan: SOMATOM Force, a dual-source CT scanner with single energy (Siemens Healthcare, Erlangen, Germany), and SOMATOM Definition Edge, a single-source CT scanner with single energy (Siemens Healthcare, Erlangen, Germany). All patients were placed in a supine position and scanned from the base of the lung to the foot. We performed pre-contrast, arterial phase, and venous phase scans. Iopromide (Ultravist 370; Bayer Korea, Seoul, Republic of Korea) was used as the contrast agent, and 2 mL/kg body weight was injected intravenously at a rate of 4 mL/s through the antecubital vein using an automatic power injector (Medrad stellant CT injector, Warrendale, PA, USA; Flowsens, Guerbet, Villepinte, France). The bolus tracking method obtained the arterial phase by setting the region of interest at the descending thoracic aorta. CT was initiated 15 s after reaching 100 Hounsfield units (HU). To perform CT venography aimed at enhancing the deep and superficial veins, CT scanning was initiated 180 s after injecting the contrast agent. Automatic exposure control (Caredose 4D, Siemens Healthcare) was activated to decrease the radiation dose. However, automatic tube potential modulation (Care KV, Siemens Healthcare) was not switched on. CT images were sent from our CT scanner to Syngo.via (Siemens Healthcare, Erlangen, Germany) to produce volume-rendering images, maximum intensity projection (MIP) with calcification, and MIP with calcification removal. The scanning parameters were as follows: tube voltage, 100 kVp; collimation, 192 × 0.6 mm (SOMATOM Force, Siemens Healthcare, Erlangen, Germany), 128 × 0.6 mm (SOMATOM Definition Edge, Siemens Healthcare, Erlangen, Germany); rotation speed, 1 s (SOMATOM Force), 0.5 s (SOMATOM Definition Edge); pitch, 0.7 (SOMATOM Force), 0.5 (SOMATOM Definition Edge); reconstruction thickness, 5 mm.

### 2.3. Qualitative Image Analysis

Two radiologists with 10 and 12 years of experience in vascular imaging, who were blinded to any clinical information except leg swelling, retrospectively reviewed the CT findings. Discrepancies were resolved by consensus. The two readers analyzed the following CT features: (a) extent of swelling (assessed by anatomical region: thigh, lower leg, and foot), (b) laterality (classified as unilateral or bilateral involvement), (c) skin thickening (direct visualization of thickened dermis on axial CT images), (d) subcutaneous edema (presence of increased attenuation and a diffuse reticular or stranding appearance within the subcutaneous fat layer), (e) pathological findings of the inguinal lymph nodes (LN) on affected side including LN enlargement, obliteration of the fat hilum of LN, perinodal fat stranding, (f) prominent enhancement of inguinal LN (observed on contrast-enhanced images), (g) the presence or absence of early venous return on MIP (defined as contrast enhancement of deep veins (e.g., femoral or popliteal) observed during the arterial or early venous phase on MIP images), and (h) presence or absence of severe arteriosclerosis obliterans. All imaging findings were systematically recorded and analyzed to evaluate their diagnostic significance for cellulitis.

### 2.4. Data Collection

Data regarding age, sex, medical history, and laboratory results, including WBC, CRP, and ESR were collected. We reviewed whether a recent event (such as local trauma) could have caused cellulitis.

### 2.5. Statistical Analysis

Continuous variables are expressed as mean ± standard deviation (SD). The student *t*-test and chi-square test were used for comparison. Sensitivity was evaluated for each imaging feature. To assess the diagnostic performance of each imaging finding, sensitivity and 95% confidence interval (CI) were calculated. Additionally, a series of pairwise chi-square tests were performed to investigate the interrelationships between CT imaging features. The analysis included five binary CT findings commonly observed in cellulitis: skin thickening, subcutaneous edema, early venous return on MIP, lymph node pathology, and prominent lymph node enhancement. Differences were considered statistically significant if the *p* value was <0.05. All statistical analyses were performed using IBM SPSS Statistics for Windows version 29 (IBM Corp., Armonk, NY, USA).

## 3. Results

### Demographics

Overall, 174 patients diagnosed with cellulitis were enrolled in this study. Among them, 112 (64.4%) were men, and 62 (35.6%) were women (age range, 16–97 years; mean age, 64.7 ± 15.4 years). A recent event that could have caused cellulitis was identified in 72 (41.4%) patients. Patient demographics are summarized in Table 1.

The mean WBC count was 11.1 ± 5.4 × 10^3^/μL in the total cellulitis population, with no significant difference between Group I (11.4 ± 5.6 × 10^3^/μL) and Group II (10.1 ± 4.7 × 10^3^/μL). The CRP level was higher in Group I (12.2 ± 9.6 mg/L) compared to Group II (9.4 ± 9.1 mg/L); however, the difference was not statistically significant. Similarly, the ESR showed no significant difference between the groups, with a mean value of 77.5 ± 37.4 mm/h in the total population (76.7 ± 37.6 mm/h in Group I vs. 80.3 ± 36.9 mm/h in Group II). The laboratory findings of the total cellulitis population and subgroup comparisons between Groups I and II are summarized in Table 2.

**Figure 2:**
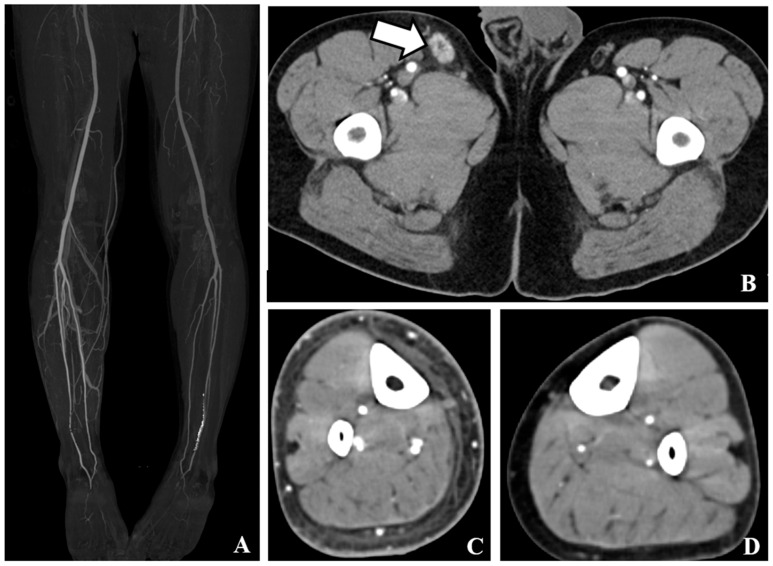
CT findings of cellulitis. (**A**). Maximum intensity projection images show early venous return at right lower limb. (**B**). Contrast-enhanced axial computed tomography (CT) showed prominent inguinal lymph node enhancement with enlargement of the right inguinal area. Slight perinodal fat infiltration is noted (white arrows). (**C**). Contrast-enhanced axial CT tomography revealed diffuse skin thickening and subcutaneous edema in the right lower leg. (**D**). Contrast-enhanced axial CT scan shows no abnormal finding at left lower leg.

We evaluated the sensitivity of various CT findings to diagnose cellulitis in all patients. Among the examined findings, subcutaneous edema exhibited the highest sensitivity (94.8%), followed by skin thickening (83.9%) and venous early return on MIP (82.8%). Pathological findings and prominent lymph node enhancement demonstrated lower sensitivities of 72.4% and 79.3%, respectively. In Group I, subcutaneous edema exhibited the highest sensitivity (97.7%), followed by skin thickening (93.1%). Early venous return on MIP (86.9%) and lymph node enhancement (80.0%) were highly sensitive, whereas lymph node enlargement had the lowest sensitivity (72.3%). In group II, subcutaneous edema showed the highest sensitivity (86.4%), followed by lymph node enhancement (77.3%) and lymph node enlargement (72.7%). The early venous return on MIP had a sensitivity of 70.5%, whereas skin thickening exhibited the lowest sensitivity (56.8%) in this subgroup. The sensitivities and corresponding 95% CI are summarized in Table 3. Representative CT findings of cellulitis include diffuse skin thickening, subcutaneous edema, early venous enhancement on MIP images, and pathological findings of inguinal LN with prominent enhancement, as shown in Figure 2.

Pairwise analysis of CT findings revealed significant associations between skin thickening and subcutaneous edema, skin thickening and early venous return on MIP, and early venous return on MIP and lymph node pathology. No significant associations were observed between skin thickening and lymph node pathology, skin thickening and lymph node enhancement, subcutaneous edema and early venous return on MIP, subcutaneous edema and lymph node pathology, or between subcutaneous edema and lymph node enhancement. The results are summarized in Table 4.

## 4. Discussion

Cellulitis is a common bacterial infection of the skin and subcutaneous tissues, presenting with localized erythema, warmth, swelling, and tenderness and often requiring prompt antibiotic therapy to prevent complications, such as abscess formation, necrotizing fasciitis, or systemic spread. Although clinical diagnosis is straightforward in many cases, imaging plays a crucial role in differentiating cellulitis from other causes of lower extremity swelling, such as DVT, lymphedema, or inflammatory arthritis [10]. MRI is particularly useful in detecting deep tissue involvement and early signs of complications, such as abscess or necrotizing fasciitis, showing diffuse subcutaneous hyperintensity on T2-weighted images and enhancement on post-contrast sequences [10,16,17,18]. Ultrasound is widely used as a first-line imaging modality due to its accessibility, and may reveal subcutaneous thickening, increased echogenicity of fat, and hyperemia on Doppler studies, although it may be limited in obese patients or in assessing deep extension [10,13,14,15]. CT is frequently used to evaluate the extent of infection, associated vascular or lymphatic involvement, and the presence of complications. However, variations in the imaging characteristics of cellulitis based on the anatomical location remain underexplored. This study aimed to evaluate the characteristic CT findings associated with cellulitis and to assess the differences in CT findings between cellulitis involving the thigh/calf (proximal cellulitis) and cellulitis confined to the foot (distal cellulitis), providing insights into potential regional variations in disease presentation.

In our study, subcutaneous edema was the most sensitive CT finding for cellulitis (94.8%), followed by skin thickening (83.9%). This finding highlights the role of subcutaneous fat involvement as a key radiological feature of cellulitis, reflecting an inflammatory response in the dermis and subcutaneous tissues. Previous studies have emphasized that subcutaneous edema is a hallmark of cellulitis resulting from increased vascular permeability and interstitial fluid accumulation in response to infection. The high sensitivity of this finding suggests that assessing subcutaneous tissue changes on CT is crucial for the radiological diagnosis of cellulitis. Despite variations in severity, subcutaneous edema remained consistently prevalent across all cases, supporting its utility as a reliable diagnostic feature. This mechanism has traditionally been explained by the classical Starling principle, which describes the movement of fluid across capillaries based on the balance of hydrostatic and oncotic pressures. However, recent research has demonstrated that this model is no longer sufficient to fully explain interstitial fluid dynamics, particularly in peripheral tissues such as the lower extremities [21,22]. Emerging evidence highlights the pivotal role of the endothelial glycocalyx layer and the lymphatic system in maintaining fluid homeostasis. Disruption of the glycocalyx and impaired lymphatic drainage contribute significantly to persistent interstitial fluid accumulation in cellulitis. Thus, the pathogenesis of subcutaneous edema should be understood within the context of both vascular permeability and lymphatic insufficiency.

In our study, early venous return on MIP in patients with cellulitis was observed in 82.8% of patients. Early venous return is not a well-established CT finding in cellulitis, making this an intriguing result of our study. This phenomenon may be attributed to localized vascular changes in response to infection. Previous research has demonstrated that inflammation induces endothelial dysfunction and vasodilation, leading to altered venous hemodynamics [23,24]. In cellulitis, this manifests as venous congestion and increased venous filling, potentially explaining the observed early venous returns. Previous studies have suggested that early venous enhancement on lower extremity CT angiography, previously considered an artifact, may reflect an active inflammatory response in patients with extensive skin infections or ischemic ulcers [25]. Byeon et al. [25] demonstrated that early venous enhancement is associated with favorable clinical outcomes and healthier vascular responsiveness, potentially serving as an imaging marker for inflammation-induced hyperemia or endothelial reactivity. These results suggest that early venous return could serve as a valuable imaging marker for cellulitis, particularly for differentiating it from other causes of lower extremity swelling. Future studies with larger cohorts and perfusion imaging may help further elucidate the clinical significance of this finding.

When comparing Groups I and II, significant differences were observed in multiple CT features. Skin thickening and subcutaneous edema were significantly less frequent in foot-only cellulitis (93.1% vs. 56.8% and 97.7% vs. 86.4%, respectively), suggesting that proximal cellulitis was associated with more pronounced inflammatory changes. Additionally, early venous return on MIP was lower in foot-only cellulitis (86.9% vs. 70.5%), and ASO was more commonly observed in foot-only cellulitis (9.2% vs. 29.5%), suggesting that ASO may contribute to altered venous hemodynamics. A previous study established that ASO leads to diminished arterial inflow and impaired perfusion in the distal lower extremities, consequently affecting venous return [26]. Given that ASO was significantly more prevalent in foot cellulitis cases in our study, it is plausible that reduced distal perfusion contributes to impaired early venous return. These findings highlight the potential interplay between arterial insufficiency and venous hemodynamics in the pathophysiology of cellulitis.

Inguinal lymph node abnormalities are frequently observed in patients with cellulitis, regardless of the location of the infection. This may be explained by the natural lymphatic drainage of the lower limb, as both proximal (thigh/calf) and distal (foot) infections primarily drain into the inguinal LN [27]. These findings are consistent with those of previous studies, including the study by Shin et al. [12], which demonstrated that inguinal lymph node enlargement and medullary fat obliteration are common CT features in cellulitis and may reflect reactive lymphadenopathy secondary to localized skin and soft tissue infections. Given the anatomical drainage pathway of the lower limb, inflammatory involvement of the inguinal LN is a plausible and unexpected imaging finding. The recognition of these patterns may aid in differentiating cellulitis from other causes of leg swelling.

In this study, we identified significant associations between several CT findings in patients with cellulitis. Skin thickening is frequently accompanied by subcutaneous edema and early venous return to MIP, suggesting that these findings may share underlying inflammatory mechanisms or represent sequential changes in disease progression. In addition, early venous return to MIP was significantly associated with lymph node pathology, potentially reflecting a more advanced or systemically reactive state. In contrast, no significant associations were observed between skin thickening and lymph node pathology or enhancement nor between subcutaneous edema and other lymphatic or vascular findings. These findings underscore the complex interplay among imaging features in cellulitis and highlight the potential value of combined CT findings in supporting the diagnosis and evaluating disease extent.

This study has some limitations. First, the retrospective design and single-center nature of this study may have limited the generalizability of our findings. Second, the lack of a non-cellulitis control group precludes assessment of the specificity and diagnostic accuracy of CT findings; however, the focus of this study was not diagnostic performance but rather the characterization of CT features in confirmed cellulitis cases. Third, the interpretation of imaging features such as lymph node enhancement or early venous return may be subject to reader variability and dependent on technical parameters such as contrast timing. Additionally, histopathological correlations of lymph node findings were not available. Furthermore, the use of MIP images as imaging protocols in this study may not reflect standard clinical practice, as most cellulitis cases are typically assessed using non-contrast CT or routine contrast-enhanced CT. While early venous return on MIP provides valuable vascular and venous information, the modality itself may influence the visibility of certain findings and potentially affect their interpretation. Finally, the distinction between Group I and Group II in this study was somewhat arbitrary, as it was based on clinical observations and a hypothesized difference in underlying conditions, such as diabetes mellitus and ASO, between the foot-only cellulitis group and the thigh or lower leg cellulitis group. This grouping may have introduced potential bias and should be interpreted with caution. Moreover, the observed differences in skin thickening and subcutaneous edema between the two groups may have been influenced by anatomical variations. The lower leg and thigh regions typically contain more subcutaneous tissue compared to the foot, potentially exaggerating the visibility of these findings in Group I. Additionally, cases with more extensive or severe cellulitis were more prevalent in Group I, which could have contributed to the perception bias regarding these imaging features. Future prospective multicenter studies with standardized imaging protocols and appropriate control groups are warranted to validate and expand upon these findings.

## 5. Conclusions

Our study highlights key differences in the CT characteristics of cellulitis depending on the anatomical location. Proximal cellulitis is associated with more pronounced inflammatory changes in the skin and subcutaneous tissues, whereas foot-only cellulitis has a stronger correlation with underlying arterial diseases. These findings underscore the importance of considering regional variations in cellulitis presentation when interpreting imaging results and making clinical decisions.

## Figures and Tables

**Table 1:** Baseline characteristics of the study population.

Characteristic	Total (n = 174)
Age	64.1 ± 15.8
Sex	
Male	112 (64.4)
Female	62 (35.6)
Underlying disease	
None	28 (16.1)
Diabetes mellitus	96 (55.2)
Hypertension	94 (54)
End-stage renal disease	21 (12.1)
Dyslipidemia	19 (10.9)
Cerebrovascular accident	15 (8.6)
Malignancy	14 (8)
Liver cirrhosis	11 (6.3)
Heart failure	9 (5.2)
Chronic obstructive pulmonary disease	5 (2.9)
Multiple comorbidities (≥2 diseases)	87 (50)
History of trauma associated with cellulitis	72 (41.4)

**Table 2:** Comparison of laboratory findings between cellulitis groups.

	Total	Group I	Group II	*p* Value
WBC (10^3^/μL)	11.1 ± 5.4	11.4 ± 5.6	10.1 ± 4.7	0.172
CRP (mg/L)	11.5 ± 9.6	12.2 ± 9.6	9.4 ± 9.1	0.107
ESR (mm/h)	77.5 ± 37.4	76.7 ± 37.6	80.3 ± 36.9	0.591

Abbreviations: WBC, white blood cell; CRP, C-reactive protein; ESR, erythrocyte sedimentation rate.

**Table 3:** Comparison of CT findings in diagnosing cellulitis between Group I and II.

CT Findings	Total (n = 174)	Group I (n = 130)	Group II (n = 44)	*p* Value
Skin thickening	83.9 [78.2–89.1]	93.1 [88.5–96.9]	56.8 [43.2–70.5]	<0.001
Subcutaneous edema	94.8 [91.4–97.7]	97.7 [94.6–100]	86.4 [75.0–95.5]	<0.001
Venous early return on MIP	82.8 [77.0–87.9]	86.9 [80.8–92.3]	70.5 [56.8–84.1]	0.012
Lymph node pathologic finding	72.4 [65.5–78.7]	72.3 [64.6–80.0]	72.7 [59.1–86.4]	0.957
Lymph node enhancement	79.3 [72.9–85.0]	80.0 [73.1–86.9]	77.3 [63.6–88.6]	0.699
Arteriosclerosis obliterans	25 (14.4)	12 (9.2)	13 (29.5)	<0.001

Abbreviations: CT, computed tomography; MIP, maximum intensity projection.

**Table 4:** Pairwise comparison of CT findings among patients diagnosed with cellulitis.

CT Features 1	CT Features 2	*p* Value
Skin thickening	Subcutaneous edema	<0.001
Skin thickening	Venous early return on MIP	0.011
Skin thickening	Lymph node pathology	0.165
Skin thickening	Lymph node enhancement	0.611
Subcutaneous edema	Venous early return on MIP	0.187
Subcutaneous edema	Lymph node pathology	0.263
Subcutaneous edema	Lymph node enhancement	0.395
Venous early return on MIP	Lymph node pathology	0.001
Venous early return on MIP	Lymph node enhancement	0.011
Lymph node pathology	Lymph node enhancement	<0.001

## Data Availability

The dataset generated and/or analyzed during the current study is available from the corresponding author upon reasonable request.

## Abbreviations

CT, Computed Tomography; MIP, Maximum Intensity Projection; LN, Lymph Node; WBC, White Blood Cell; CRP, C-Reactive Protein; ESR, Erythrocyte Sedimentation Rate; SD, Standard Deviation; CI, Confidence Interval.
